# A double-blind placebo-controlled, randomised study comparing gemcitabine and marimastat with gemcitabine and placebo as first line therapy in patients with advanced pancreatic cancer

**DOI:** 10.1038/sj.bjc.6600446

**Published:** 2002-07-02

**Authors:** S R Bramhall, J Schulz, J Nemunaitis, P D Brown, M Baillet, J A C Buckels

**Affiliations:** Department of Surgery, Liver Unit, Queen Elizabeth Hospital, Birmingham B15 2TH, UK; US Oncology, 895 Middleground Blvd, Newport News, Virginia, USA; US Oncology, 3535 Worth Street, Dallas, Texas, USA; British Biotech Pharmaceuticals Ltd., Oxford, UK

**Keywords:** marimastat, gemcitabine, pancreatic cancer

## Abstract

Pancreatic cancer is the fifth most common cause of cancer death in the western world and the prognosis for unresectable disease remains poor. Recent advances in conventional chemotherapy and the development of novel ‘molecular’ treatment strategies with different toxicity profiles warrant investigation as combination treatment strategies. This randomised study in pancreatic cancer compares marimastat (orally administered matrix metalloproteinase inhibitor) in combination with gemcitabine to gemcitabine alone. Two hundred and thirty-nine patients with unresectable pancreatic cancer were randomised to receive gemcitabine (1000 mg m^−2^) in combination with either marimastat or placebo. The primary end-point was survival. Objective tumour response and duration of response, time to treatment failure and disease progression, quality of life and safety were also assessed. There was no significant difference in survival between gemcitabine and marimastat and gemcitabine and placebo (*P*=0.95 log-rank test). Median survival times were 165.5 and 164 days and 1-year survival was 18% and 17% respectively. There were no significant differences in overall response rates (11 and 16% respectively), progression-free survival (*P*=0.68 log-rank test) or time to treatment failure (*P*=0.70 log-rank test) between the treatment arms. The gemcitabine and marimastat combination was well tolerated with only 2.5% of patients withdrawn due to presumed marimastat toxicity. Grade 3 or 4 musculoskeletal toxicities were reported in only 4% of the marimastat treated patients, although 59% of marimastat treated patients reported some musculoskeletal events. The results of this study provide no evidence to support a combination of marimastat with gemcitabine in patients with advanced pancreatic cancer. The combination of marimastat with gemcitabine was well tolerated. Further studies of marimastat as a maintenance treatment following a response or stable disease on gemcitabine may be justified.

*British Journal of Cancer* (2002) **87**, 161–167. doi:10.1038/sj.bjc.6600446 www.bjcancer.com

© 2002 Cancer Research UK

## 

Pancreatic cancer is the fifth most common cause of cancer death in the western world. There are an estimated 26 000 deaths in the US and 50 000 deaths per year in Europe (excluding the former USSR) (Fernandez *et al*, 1994; Wingo *et al*, 1995). The overall 5 year survival rate (5YSR) for patients with pancreatic cancer ranges from <1% to less than 5% even in the best prognosis patients and there has been little improvement in survival in the last 20 years (Bramhall *et al*, 1995). The 5YSR for patients with pancreatic cancer is the lowest reported for any cancer (Ries *et al*, 1994).

Recent changes in attitude towards pancreatic cancer treatment have led to the development of novel agents for the treatment of this disease. Several studies have reported encouraging results with single chemotherapy agents or combination treatments (Mallinson *et al*, 1980; Casper *et al*, 1994; Palmer *et al*, 1994; Carmichael *et al*, 1995; Burris *et al*, 1997) and gemcitabine has been compared to 5-fluorouracil (5-FU) in a phase III study in patients with advanced pancreatic cancer (Burris *et al*, 1997). The results of this study suggested that patients receiving gemcitabine had both an improved survival and patient benefit compared to those patients receiving 5-FU and these data and data from several other studies have led to the widespread acceptance of gemcitabine as first-line therapy in patients with advanced pancreatic cancer (Casper *et al*, 1994; Carmichael *et al*, 1995; Rothenberg *et al*, 1996; Burris *et al*, 1997; Storniolo *et al*, 1999).

The rapid increase in knowledge of the molecular and cellular biology of malignancy has enabled scientists to accurately target cellular pathways with synthetic compounds and inhibit these pathways for potential therapeutic benefit. Several of these strategies have been tested in clinical trials in patients with a variety of tumour types. One such treatment strategy has been the inhibition of matrix metalloproteinases (MMPs).

The MMPs are proteolytic enzymes that each have different substrate specificities within the extracellular matrix and have been shown to be important in its degradation (Cottam and Rees, 1993). An imbalance between activated MMP and tissue specific inhibitors is believed to lead to extracellular matrix degradation and tumour invasion. Studies in animal models of malignancy have reported that MMP inhibitors (MMPIs) can restrict the growth and regional spread of solid tumours, inhibit metastases and inhibit tumour neovascularisation (Brown and Giavazzi, 1995). Expression of several members of the MMP family is significantly greater in pancreatic cancer than normal pancreas (Sato *et al*, 1994; Gress *et al*, 1995; Bramhall *et al*, 1996, 1997).

Several broad-spectrum synthetic MMPIs have been developed and marimastat (British Biotech Pharmaceuticals Ltd., Oxford, UK) was the first in a series of new generation MMPIs with sufficient oral absorption to justify its use in clinical trials. Marimastat and its predecessor batimastat have been widely tested in a range of cancer models (Davies *et al*, 1993; Chivri *et al*, 1994; Watson *et al*, 1995, 1999). The principal effect of marimastat is to retard tumour growth and metastatic spread but it does not display cytotoxic activity in cell culture and no tumour regression has been observed in animal models (Chivri *et al*, 1994; Watson *et al*, 1995; Zervos *et al*, 1997, 1999). The theoretical role of marimastat therefore, would be as a maintenance therapy with or without concomitant cytotoxic therapy, in patients at risk of relapse following ‘curative’ therapy or with minimal or microscopic disease. The activity of marimastat as a novel agent has first been determined in patients with advanced disease.

A phase II study of marimastat in patients with advanced pancreatic cancer determined an appropriate dose schedule that achieved acceptable levels of toxicity. The main toxicity noted with marimastat was a dose dependent musculoskeletal pain that responded to drug omission but if treatment persisted could lead to contractures, particularly of the hand. The same study demonstrated a surrogate clinical response determined by a decrease in the tumour antigen CA19-9 production. Both toxicity and tumour antigen effect appeared to be dose dependent (Nemunaitis *et al*, 1998; Evans *et al*, 2001). A pivotal international multi-center randomised study compared the effect of three different doses of marimastat with gemcitabine in patients with advanced pancreatic cancer. The study failed to reach its primary endpoint but did show a dose-dependent effect of marimastat and reported a 1-year survival of 19% for patients treated with gemcitabine and 20% for patients receiving 25 mg b.i.d of marimastat (*P*=ns) (Bramhall *et al*, 2001b). Progression free survival was significantly better for patients treated with gemcitabine (*P*=0.0001) but this was predicted based on the mode of action of the two drugs. Exploratory analysis of these data suggested that survival in patients treated with marimastat with non-metastatic pancreatic cancer was significantly better than in patients with metastatic disease (*P*=0.035) but this was not the case in patients treated with gemcitabine (*P*=0.456) (Bramhall *et al*, 2001b). These data suggest that marimastat might be more beneficial in patients with low volume disease and this was also supported in a placebo-controlled study of marimastat in gastric and gastro-oesophageal cancer (Bramhall *et al*, 2001a).

The mode of action of marimastat and the toxicity profile determined from the phase II and III studies suggest that marimastat might have a role in combination with a cytotoxic agent. Data from a phase I study indicated that the combination of gemcitabine and marimastat is well tolerated (unpublished data). This international multi-centre randomised study compares the effect of gemcitabine combined with marimastat against gemcitabine and placebo on survival, safety, time to disease progression, time to treatment failure, radiological response and duration of response.

## PATIENTS AND METHODS

### Patient population

This randomised study included patients with histologically or cytologically proven adenocarcinoma of the pancreas that was unresectable on computerised tomographic (CT) or magnetic resonance imaging (MRI). Patients were required to be aged over 18 years and have a Karnofsky performance status (KPS) of at least 60%. Patients had to have adequate bone marrow reserve at study entry, defined as an absolute granulocyte count of ⩾1500 mm^3^, platelet count of ⩾100 000 μl^−1^ and haemoglobin ⩾10 gm dL^−1^. Adequate baseline hepatic function (defined as bilirubin <2.0 mg dL^−1^, aspartate transaminase (AST), alanine transaminase or alkaline phosphatase ⩽five times the upper limit of normal) and adequate renal function (creatinine ⩽1.5 mg dL^−1^) were also required. Any form of previous systemic anti-cancer therapy as a primary intervention for locally advanced or metastatic disease was disallowed, as was prior exposure to a metalloproteinase inhibitor or gemcitabine. Patients who had received prior adjuvant or consolidation chemotherapy or radiotherapy and relapsed within 6 months of finishing therapy were excluded. Pregnant or lactating patients were excluded. Patients who had received other investigational agents within 4 weeks prior to commencing the study were excluded. All tumours were staged using the UICC TNM classification and then stage grouped according to the American Joint Committee on Cancer Staging criteria for pancreatic cancer.

The primary study endpoint was overall survival. Secondary study endpoints were objective tumour response rate, duration of response, time to treatment failure, time to disease progression and quality of life assessment. Safety and tolerability were also assessed. The study was performed in accordance with the Declaration of Helsinki, approved by Institutional Review Boards and local regulatory authorities as appropriate and conducted in accordance with the FDA Guideline on Good Clinical Practice.

### Patient assignment

Signed and witnessed informed consent was obtained from each patient prior to study entry. Patients were assigned to study treatment using a computer generated random code according to the method of minimisation. This method balanced the treatment groups on the basis of stage of disease (stage I/II, III or IV), KPS (60–70% *vs* 80–100%), gender, disease status (newly diagnosed *vs* recurrent *vs* recurrent + other treatment), measurable disease (measurable *vs* non-measurable) and study centre. Patients were randomised to receive either 1000 mg m^−2^ of gemcitabine hydrochloride by intravenous infusion and marimastat 10 mg b.i.d or gemcitabine at the same dose and placebo. The marimastat/placebo treatment was administered in a double-blinded fashion.

### Treatment

Patients received marimastat or placebo with food. The dose of marimastat could be reduced if musculoskeletal or other toxicities developed. If musculoskeletal toxicities were greater than or equal to National Cancer Institute – Common Toxicity Criteria (NCI CTC) grade 2 or other toxicity of grade 4 developed, marimastat was omitted until the symptoms had abated. Patients could then restart at a 50% dose reduction i.e. once daily instead of twice-daily administration. If toxicity of the severity described above recurred, then marimastat again would be omitted until the symptoms had abated and a further 50% dose reduction would be instituted i.e. alternate day dosing. If symptoms still persisted then consideration to withdraw the patient was made. Once a marimastat dose reduction had been mandated, no escalation to the previous level was permitted at a later date. Patients were seen on a weekly basis while receiving gemcitabine and on a monthly basis if receiving marimastat/placebo alone and after 28 days following study discontinuation.

Gemcitabine hydrochloride (Gemzar® Eli Lilly and Company, Indianapolis, USA) was supplied as a lyophilised powder. The drug was stored and prepared in accordance with the manufacturer's instructions. Patients were seen and administered 1000 mg m^−2^ weekly for the first 7 weeks with a rest in week eight and thereafter 1000 mg m^−2^ weekly for 3 weeks, with a rest in the fourth week. A dose reduction of 25% was permitted for granulocyte counts of 0.5–0.99 μl^−1^ or a platelet count of 50 000–99 999 μl^−1^ and if the counts were lower then the next dose was omitted. Patients who could not be treated for 6 weeks due to toxicity would be withdrawn from the study. Gemcitabine dose was recalculated if patients experienced a change in weight of >10%.

Patients were not allowed to receive concomitant anti-cancer therapy.

### Statistical analysis

The sample size of 200 (100 per group) was calculated to enable detection of absolute differences in survival at 18 months of 13.5% between those patients treated with gemcitabine and marimastat and those treated with gemcitabine and placebo, with a power of ⩾80% and using a significance level of 0.05 (log-rank test). These calculations were based on 90% mortality at study censure with gemcitabine and placebo and a mortality of 76.5% in the gemcitabine and marimastat treated group. The treatment groups were compared on an intention-to-treat basis using Kaplan-Meier survival curves. In all survival analyses, patients who were lost to follow up were censored at last known date alive. Proportions were tested using the χ^2^ test. Patient benefit data was tested using the Wilcoxon rank-sum test, and repeated measures analysis was applied to the quality of life data.

### Efficacy and safety evaluation

The primary efficacy endpoint in this study was survival. All survival analyses were performed on an intention-to-treat basis and included all patients minimised. Treatment continued until death, disease progression or drug toxicity that warranted removal from the study. Once patients progressed, they were removed from the study and received best supportive care as determined by the investigator. If a patient was removed from the study for any reason, they were seen 1 month later and thereafter every 2 months until death.

Secondary endpoints were objective tumour response rate, duration of response, time to treatment failure, time to disease progression, quality of life assessment and safety and tolerability. Objective tumour response rate was defined according to the WHO criteria for response. Consecutive chest X-ray, CT or MRI scans were reviewed by the same radiologist in the patients centre and response was defined as complete, partial, stable disease or progressive disease. CT or MRI scans were performed within the 14 days prior to day 0 and then every 2 months for the first 6 months and thereafter every 3 months and at early termination. Complete response was defined as disappearance of all known disease for a minimum of 4 weeks. Partial response was defined as a ⩾50% decrease in the sum of the products of the largest perpendicular diameters of all measurable lesions for a minimum of 4 weeks. Progressive disease was defined as a ⩾25% increase in the sum of the products of the largest perpendicular diameters of all measurable lesions from the study nadir, the appearance of new lesions or death. Patients who did not meet the criteria for complete response; partial response or progressive disease and who remained on study for at least 4 weeks were classified as having stable disease. Duration of response was defined in patients with a partial response as the time of initiation of therapy to the date of objective disease progression and in those patients with a complete response as the time from the date of onset of the response to the date of objective disease progression. Patients dying prior to documented progressive disease were considered to have experienced progressive disease at death. Time to treatment failure was defined as the time from randomisation to permanent discontinuation of the combination regimen for any reason, including death, disease progression, unacceptable toxicity, investigator decision or patient decision. Quality of life assessment included an assessment of pain (Memorial Pain Assessment card – MPAC), level of analgesic required, KPS, surgical interventions to alleviate cancer related symptoms, patient weight changes and formal quality of life assessment (using FACT-Pa questionnaire).

Patients were evaluated by weekly examination of vital signs, weight and full blood counts and 4-weekly history, clinical examination and biochemical profile. KPS, FACT-Pa quality of life assessment, pain assessment and analgesic rating were performed at baseline and every 4 weeks thereafter. All signs, symptoms and laboratory abnormalities were assessed using the NCI CTC criteria for toxicities. In addition, a specific rating for grading musculoskeletal toxicity was developed for use with marimastat (Bramhall *et al*, 2001b). Grade 1 musculoskeletal toxicity was defined as aches and pains with no restriction of activity. Grade 2 was defined as having pain causing restriction of activity. Grade 3 was defined as having pain and the presence of nodules or clinically inflamed joints or tendons and grade 4 was pain and the presence of a contracture.

## RESULTS

Two hundred and thirty-nine patients were recruited from 18 North American (114 patients) and 19 European (125 patients) sites between September 1997 and April 1998. Of the 239 patients randomised 120 were randomised to receive gemcitabine and marimastat (GM) and 119 to receive gemcitabine and placebo (GP). Forty-four patients received chemotherapy post study (21 in the GM and 23 in the GP groups). All patients are included in this intention-to-treat analysis, but two patients randomised to GM and two randomised to GP treatment did not receive any study drug and so are excluded from the safety analysis but not the efficacy analysis. The patient demographics and key prognostic factors were similar between the two treatment groups (Table 1

**Table 1:**
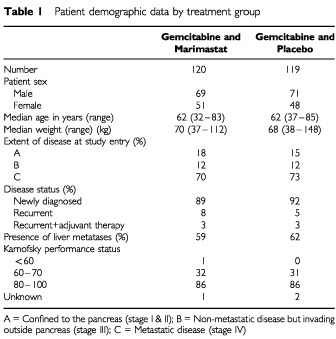
Patient demographic data by treatment group

).

**Figure 1:**
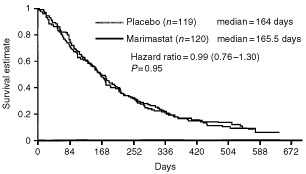
Primary mortality analysis (intention-to-treat).

Final analysis of the study was performed when 90% mortality had occurred in the GM treatment group (August 1999). Differences between individual treatment groups were determined using the log-rank test. Analysis revealed no difference in overall survival between the two treatment arms (*P*=0.95, hazard ratio (HR) 0.99, 95% confidence intervals (CI) 0.76–1.30). There was no difference in median survival, GM (165.5 days) compared to GP (164 days) (Figure 1

). The 1-year survival was 18% for the GM group and 17% for the GP group.

There was no significant difference in response rates between the two treatment arms (Table 2

**Table 2:**
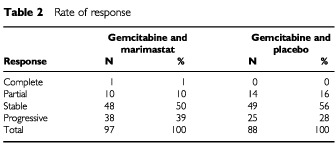
Rate of response

), however, there was a trend in favour of placebo in the duration of response with a median of 118 days in the GM arm (*n*=14) and 258 days (*n*=15) in the GP arm (*P*=0.07 log-rank test). The median time to treatment failure favoured the GM treated patients, 107 days compared to 89 days in the GP treated patients (*P*=0.70) (Figure 2

**Figure 2:**
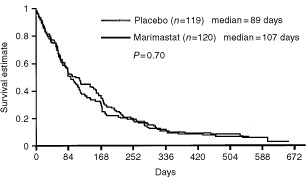
Time to treatment failure.

). Analysis of progression-free survival however, revealed no difference between the GM arm (92.5 days) and the GP arm (96 days) (*P*=0.68, HR 0.95, 95% CI 0.73–1.23).

Exploratory analyses of overall survival in patients with metastatic (stage IV) and non-metastatic (stage I/II/III) disease revealed no difference between the GM and GP treatment arms. The median survival time in patients without metastases and treated with GM (*n*=36) was 266 days and in those treated with GP (*n*=32) was 290 days (*P*=0.67, HR 1.13, 95% CI 0.66–1.94). The median survival time in patients with metastatic disease and treated with GM (*n*=84) was 138.5 days and in those treated with GP (*n*=87) was 140 days (*P*=0.59, HR 0.92, 95% CI 0.67–1.25). Further exploratory analysis of the subset of patients with a good KPS (80–100) and with disease confined to the pancreas gland (stage I/II) revealed an improved survival in those patients treated with GM (*n*=14, median survival time=451 days) when compared with those patients treated with GP (*n*=16, median survival time=266.5 days) (*P*=0.33, HR 1.54, 95% CI 0.65–3.66).

There were no statistically significant differences between GP and GM treated arms with respect to pain, mood, analgesic use, KPS or weight as assessed by change from baseline standardised areas under the curve (AUCs) up to 2 months (Bailey *et al*, 1998). There was however, a difference in quality of life assessed using FACT-Pa. The median change from baseline standardised AUC to 2 months was 0.88 for GP treated patients and −1 for GM treated patients (*P*=0.048, Wilcoxon rank sum test), indicating an improved quality of life for GP treated patients.

### Safety

Compliance and tolerance of therapy was good with patients receiving a median of 106 days (range 0–654 days) treatment with marimastat in the GM arm and 85 days (range 0–619 days) treatment with placebo in the GP arm. Gemcitabine treatment continued for a median of 11 doses (range 0–56 doses) and 10 doses (range 0–56 doses) in the GM and GP arms respectively. Overall 2.5% (three out of 118) of patients treated with marimastat were withdrawn due to adverse events compared to 3.4% (four out of 117) in the placebo arm; 0.8% (one out of 118) of GM patients and 1.7% (two out of 117) GP patients were withdrawn due to possible gemcitabine related toxicity. One of the marimastat patients was withdrawn due to musculoskeletal toxicity. The reasons for withdrawal due to possibly drug related events related to gemcitabine were rash, vasculitis and anaemia.

All treatment arms were relatively well tolerated and there were no deaths on study attributable to treatment. All deaths were considered to be secondary to disease progression. The NCI-CTC grades were assigned as a multiple of the normal range irrespective of causality (Table 3

**Table 3:**
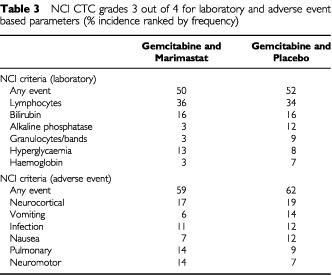
NCI CTC grades 3 out of 4 for laboratory and adverse event based parameters (% incidence ranked by frequency)

), therefore factors related to the underlying disease such as liver metastases may have an influence. The overall incidence of grade 3 or 4 adverse events was similar between the two treatment arms. As expected, gemcitabine exerted a myelosuppressive effect on all haematological parameters with no significant difference being seen between either treatment arm. Severe musculosketal toxicities were reported with both marimastat and placebo, although marginally more commonly in the GM treatment arm (Table 4

**Table 4:**
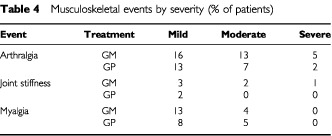
Musculoskeletal events by severity (% of patients)

). Musculoskeletal adverse events were reported in 59% of GM treated patients and 44% of GP treated patients (*P*=0.031, χ^2^ test).

GM patients (47.5%) and GP patients (38.6%) had their marimastat/placebo dosage reduced or required a drug holiday because of musculosketal toxicity. The median time to a musculoskeletal event was shorter in the GM arm (77 days) compared with the GP arm (139 days) (*P*=0.053, log-rank test). The mean number of gemcitabine dose reductions was 2.61 in the GM patients and 2.19 in the GP patients.

## DISCUSSION

The prognosis in patients with pancreatic cancer is poor. Bramhall *et al* (1995) reported an overall 5YSR in a series of 13 560 patients with pancreatic cancer of <0.2% with only 2.6% undergoing resection (5YSR of 5.5%) (Bramhall *et al*, 1995). Gudjonsson (1987) reported similar results in an overview of the published literature and an analysis of patients from his own institution. Traditionally therefore, most clinicians have viewed pancreatic cancer in a nihilistic manner and have been only prepared to offer supportive care (Douglass, 1987). Until recently there had been little data to contradict this approach in patients with advanced disease, with the majority of studies failing to report any survival advantage in patients treated with conventional chemo-radiotherapy (Bramhall and Neoptolemos, 1997). The recent introduction of gemcitabine however, has lead to the widespread acceptance of this drug as first line treatment for patients with advanced pancreatic cancer and attention has now turned to the combination of gemcitabine with other therapies (van Moorsel *et al*, 1997; Colucci *et al*, 1998; Cardenal *et al*, 1999; Cascinu *et al*, 1999; Hidalgo *et al*, 1999).

There has been a recent increase in the development of novel classes of agents specifically directed towards molecular ‘defects’ in malignant disease and the MMPIs were the first class of such agents to enter clinical trials (Bramhall *et al*, 2001a,b). The tumour biology of pancreatic tumours made patients with pancreatic cancer an obvious choice to test MMP inhibition as a treatment strategy and phase II and III studies of marimastat have now been completed and reported (Bramhall *et al*, 2001b; Evans *et al*, 2001). The differing mode of action and toxicity profile of gemcitabine and marimastat make the combination of these agents an obvious therapeutic strategy to investigate.

This study was powered to detect an absolute difference in survival between the GM and GP treatment arms. The primary mortality analysis did not reveal a significant difference in survival between the treatment groups.

In the randomised study of gemcitabine *vs* 5-FU, reported by Burris *et al* (1997) survival of the gemcitabine patients was very similar to the survival seen in both treatment arms of this study and in the gemcitabine and 25 mg b.i.d treated arms of the previously mentioned phase III study of marimastat *vs* gemcitabine (Bramhall *et al*, 2001b). The median survival time for patients receiving gemcitabine in the study by Burris was 169 days and 132 days for those receiving 5-FU, the median survival time for patients receiving gemcitabine in the study by Bramhall was 167 days and for those patients receiving marimastat 25 mg b.i.d was 125 days. In this study the median survival time for the GM arm was 165.5 days and 164 days in the GP arm. The 1-year survivals were 18 and 2% for gemcitabine and 5-FU (Burris *et al*, 1997), 19 and 20% for gemcitabine and marimastat (Bramhall *et al*, 2001b) and in this study 18 and 17% for GM and GP respectively. The three studies had similar patient populations but patients in the Burris study had to be symptomatic. There is, however a large imbalance in performance status between the three studies. In the Burris study 31% of patients had a performance of 70% or more, whereas patients in the study by Bramhall had had a better performance status with 75% of patients having a KPS of more than 70% and in this study 72% had a KPS of more than 70%. The efficacy results of the Burris study are not therefore really comparable with the other two studies.

There is little support for therapeutic benefit of marimastat in combination with gemcitabine in the current study. Overall survival was no different for patients treated with gemcitabine in combination with marimastat compared to gemcitabine in combination with placebo. There was a trend towards a survival difference in exploratory sub-group analysis when patients with disease confined to the pancreas and with a good performance status were treated with GM (although the numbers in this analysis were very small) and these data are consistent with the findings of the studies of marimastat in advanced pancreatic and gastro-oesophageal cancer which both suggested that marimastat was more efficacious in patients with low volume disease (Bramhall *et al*, 2001a,b).

Marimastat was generally well tolerated and appears to have an acceptable toxicity profile in combination with gemcitabine. This study confirms the findings from the two previous randomised studies of marimastat that marimastat treatment is associated with musculoskeletal pain. Although this can become severe if patients continue to take marimastat in the presence of symptoms, it does appear to reverse on cessation of treatment, in most cases within 1–2 weeks. Further evidence for this can be seen by the reduction in severe musculoskeletal complications seen in this study (5%) compared with the two previous studies (7% with 10 mg b.i.d and 12.8% with 25 mg b.i.d). The side effects of marimastat can be understood and managed by the patients themselves, unlike some of the more severe side effects associated with cytotoxic treatments.

The choice of marimastat dose in this study might have been sub-optimal when the results of the previous randomised study of marimastat in pancreatic cancer are considered. This study was designed and commenced prior to analysis of the results of the comparative study between gemcitabine and marimastat. In this study there was a dose dependent effect of marimastat with the dose of 25 mg b.i.d comparing favourably with gemcitabine in pancreatic cancer (Bramhall *et al*, 2001b). In this study marimastat dosing was 10 mg b.i.d and could be considered sub-optimal, however even in sub-group analysis there was very little indication of synergy between marimastat and gemcitabine. In conclusion the combination of gemcitabine and a MMPI can be safely delivered to patients with pancreatic cancer but there appears little evidence to support further study of this combination. However, further studies of the use of marimastat as maintenance treatment following a response or stable disease on gemcitabine may be justified on the basis of the recent findings in gastric cancer. This study found that when marimastat was compared with placebo in advanced gastric cancer prolonged survival was found to be most marked in patients who had previously received chemotherapy and in those patients without metastatic disease (Bramhall *et al*, 2001b).

## MEMBERS OF THE MARIMASTAT PANCREATIC CANCER STUDY GROUP

### North America

Dr Lee Schwartzberg, West Clinic, 1775 Moriah Woods Blvd, Suite 5, Memphis, Tennessee; West Clinic, 920 Madison, Suite 625, Memphis, Tennessee; West Clinic, 7620 Southcrest Pkwy, Suite 2, Southaven, Mississippi; Mid South Clinical Research Institute, Memphis, Tennessee, USA.

Dr Ron E Pruitt, Nashville Medical Research, 4320 Harding Rd, Suite 309W, Nashville, Tennessee; Saint Thomas Hospital, 4220 Harding Rd, Nashville, Tennessee, USA.

Dr John Nemunaitis, US Oncology, 3535 Worth Street, Dallas, Texas, USA.

Dr Ronald Peck, University of Virginia Cancer Center Charlottesville, Virginia; Culpeper Memorial Hospital, Culpeper, Virginia, USA.

Dr Adrian Langleben, Royal Victoria Hospital, 687 Pine Avenue West; Jewish General Hospital, 3755 Cote-Ste-Catherine; Montreal General Hospital, 1650 Cedar Avenue; St Mary's Hospital, 3830 Lacombe Street; Montreal, Quebec, Canada.

Dr Charles Blanke, Vandebilt University Medical Centre, Nashville, Tennessee; Nashville VA Medical Center, Nashville, Tennessee, USA.

Dr Alexandre Rosemurgy, Tampa General Hospital, Tampa, Florida; Harbourside Medical Tower, Tampa, Florida, USA.

Dr Jules Harris, Rush Medical College, Chicago, Illinois, USA.

Dr Joseph Schulz, US Oncology, Newport News, VA, USA.

Dr Allen L Cohn, University of Colorado Health Sciences Center, Denver, Colorado, USA.

Dr Paul Kaywin, Oncology/Hematology Group of South Florida, 8940 Kendall Drive, Miami, Florida; Baptist Hospital of Miami, 8900 North Kendall Drive, Miami, Florida; Lessner and Troner, 8950 North Kendall Drive, Miami, Florida, USA.

Dr Arthur Staddon, Oncology Hematology Associates, Allegheny University Hospitals, Graduate, Lombard St, Philadelphia, Pennsylvania, USA.

Dr Jose Lutzky, Mount Sinai Medical Center, Miami Beach, Florida, USA.

Dr Mukund Didolkar, Sinai Hospital of Baltimore, Baltimore, Massachusetts, USA.

Dr Hobert W Harris, San Francisco General Hospital, California, USA.

Dr Robert Winston, Hubert Humphrey Cancer Centers; Park Nicollet Clinic Cancer Center; Willmar Medical Center; Minnesota Oncology Hematology Centres; Ridgeview Medical Place; Colombia Park Clinic; and privates offices; Minnesota, USA.

Dr Irving Berkowitz, Christiana Hospital, Newark, DE; Delaware Clinical and Laboratory Physicians, Wilmington, DE; Oncology Associates, Wilmington, DE, USA.

Dr Jeffery Clark, Massachusetts General Hospital, Boston, Massachusetts, USA.

### Europe

Mr John Buckels, Queen Elizabeth Hospital, Birmingham, UK.

Dr David Dunlop, Glasgow Royal Infirmary, Glasgow, UK.

Mr Colin Johnson, Southampton General Hospital, Southampton, UK.

Prof S Van Belle, UZ Gent, Oncologisch Centrum, Belgium.

Prof Howie Scarffe, Christie Hospital, Manchester, UK.

Dr L Ledermann, Middlesex Hospital, London, UK.

Dr R Charnley, Freeman Hospital and Newcastle General Hospital, Newcastle, UK.

Prof W Steward, Leicester Royal Infirmary, Leicester, UK.

Dr R Houston, Royal Victoria, Belfast, UK.

Prof J Neoptolemos, Royal Liverpool University Hospital, Liverpool, UK.

Dr Bernd von Lampe, WBF, Berlin, Germany.

Dr Stephen Falk, Bristol Oncology Centre, Bristol, UK.

Dr Pippa Corrie, Addenbrooke's Hospital, Cambridge, UK.

Prof Malfertheiner, University of Magdeburg, Germany.

Prof Van Cutsem, University Hospital Gasthuisberg, Belgium.

Dr Colin Askill, Singleton Hospital, Swansea, UK.

Prof J Carmichael, Nottingham City Hospital, Nottingham, UK.

Dr Christos Dervenis, Agia Olga Hospital, Athens, Greece.

Mr J Reynolds, St James Hospital, Dublin, Ireland.
